# The story of ‘us’ is the story of ‘me’: A cross-sectional test of the influence of insecure attachment on narratives of romantic transgressions and high points

**DOI:** 10.1371/journal.pone.0306838

**Published:** 2024-09-06

**Authors:** Veronica M. Lamarche, Laura E. R. Blackie, Kate C. McLean

**Affiliations:** 1 Department of Psychology, University of Essex, Colchester, United Kingdom; 2 School of Psychology, University of Nottingham, Nottingham, United Kingdom; 3 Department of Psychology, Western Washington University, Bellingham, United Kingdom; Southwest University, CHINA

## Abstract

Narratives play an important role in the development of the self-identity. Romantic relationships offer a powerful context in which to develop these narratives about the self through the good and the bad experiences people have with their partners. However, the stories we tell can also be colored by how we already see ourselves. In a secondary analysis, using a prospective longitudinal study of people in established romantic relationships (*N =* 402), we tested pre-registered hypotheses regarding how attachment anxiety and avoidance lead people to develop narratives about their relationship high-points and transgressions, and whether these narratives influence their relationship satisfaction over time. Relatively higher avoidance, but not anxiety, was related to narrative construction. Those relatively higher in avoidance made more negative event connections about themselves in their transgression narratives, and more positive event connections about themselves in their relationship high-point narratives. Narrative content, however, did not mediate the association between attachment anxiety and avoidance and relationship satisfaction. Despite the lack of support for some of our pre-registered hypotheses, these findings provide valuable insights into how insecure attachment influences the stories people tell about their relationships, and how they link these events back to the self.

## Introduction

People use narratives to create meaning and make sense of the world around them [1]. Interpersonal relationships are a critical part of this narrative sense-making (e.g., [2,3]). Romantic relationships in particular provide a powerful context in which to build such narratives, as they are a central domain of identity (e.g., [4,5]), in which people make meaning of the varied interactions they have with their partners (e.g., [4,6,7]). However, not everyone will interpret the same interpersonal events in a relationship the same way. The narratives people tell about their relationships, and what it means about themselves, should be influenced by the working models people have about themselves and others [8–10]. Furthermore, these narratives should influence how people understand the quality of their relationships, and therefore their relationship satisfaction [11–13]. In the current paper we make use of a longitudinal dataset to examine narrative construction for relationship transgressions and high-points among romantically involved anxiously and avoidantly attached people, and whether the types of narratives people build can help explain the negative association between insecure attachment styles and relationship satisfaction.

### What does this say about me? narrative construction and event connections

Humans have a fundamental need to make sense of and build meaning from what would otherwise feel like random day-to-day experiences [14,15]. One way in which people impose meaning and organize these experiences is through narratives. From early mythologies attributing natural disasters to capricious gods, to a modern romantic comedy depicting the need to suffer heartbreak before finding ‘the one’, the narratives used to explain why things happen the way they do help people to understand other’s actions and what they mean to them. People engage in similar narrative story-telling about their own lives in order to understand who they are. Narrative identity [16] is developed through a process of reconstructing autobiographical past events and reflecting on the meaning of those events for one’s identity. Such reflection helps create a “through-line” in people’s lives, organizing their experiences in a way that provides a sense of cohesion, consistency, purpose and meaning ([17,18]; see also [19]). Narratives about their own lives allow people to explain to themselves and others who they are now, and what they are likely to be like in the future.

To create this narrative tapestry across autobiographical experiences, people need to be able to link or connect one event in their life with another, as well as to the self [19]. Self-event connections refer to the narrative construction of links between a past event and one’s current understanding of self [19]. When a connection is made, that event may become integrated into one’s narrative identity. These events may be good (high points) or bad (low points) autobiographical experiences. However, whether these autobiographical events confer positive or negative information about the self is not just determined by the valence of the event itself. People can see an event as having either good or bad implications for the self (i.e., make positive or negative event connections). Consider, for example, hypothetical siblings Amani and Ikenna, who both agree their parent’s tumultuous divorce was a low point in their lives but have developed different narratives about what it means for their own love lives in adulthood. For Ikenna, his parent’s divorce means that true love does not exist, and he connects this event with his inability to form lasting romantic partnerships. For Amani, her parent’s divorce means that it is very important to find a compatible life partner, and she connects this event with the high standards she expects from her partners. Thus, individual differences in narrative meaning-making are critical to understanding an individual’s identity and, in this case, their relationship.

### What does this say about us? Narrative-building in relationships

Romantic relationships offer a unique context for people to experience, and build narratives about, the world around them [4,20]. For many in the geopolitical west, romantic relationships are central to their sense of self and identity [21,22]. People also rely heavily on these bonds to help make sense and meaning of the world around them [6,7]. For example, unexpected actions by politicians [23] and fears of illness and death [24,25] motivate people to affirm their commitment and closeness in their relationships. Similarly, the epistemic desire for shared understanding leads people to communicate in a way that aligns with their partner’s beliefs, leading to greater closeness [26]. Over the course of the relationship, partners begin to develop a shared sense that they both think, feel, and believe the same things about the world around them [7]. This perceived shared understanding of the world influences their interactions with each other, and their interactions with the broader world. Thus, when the world outside of the relationship becomes uncertain and unpredictable, people turn inwards to their relationships to affirm meaning and certainty [6,23].

People also rely on narratives to make sense of their relationships ([4,27]; see also [28]). For example, measures of relationship quality (e.g., [29]) ask people to reflect on the historical timeline of their relationship to answer questions about how well their partner meets their needs and whether their relationship is better or worse than others on average (and implicitly, what does it mean for the self to exist in such a relationship). Being able to confidently affirm that a relationship is safe, and a partner is typically responsive to one’s needs is a fundamental feature of satisfying and stable relationships [30]. Indeed, relationship satisfaction captures the extent to which the positive experiences in a relationship outweigh the negatives, as well as meet or exceed expectations [12]. When a partner says something hurtful, fails to be responsive, or is unwilling to forgive or compromise, people may view these actions as indicative of who they are as a person (e.g., someone not worthy of love), as well as indicative of the relationship (e.g., a dissatisfying one). Thus, not only do autobiographical events within a relationship create a narrative that helps someone understand who they are as a person (i.e., their narrative identity) but also creates a narrative about the quality of their relationship and how satisfied they should be in it.

### What does it all mean to me? attachment style and relationship perceptions

Narratives provide an opportunity to help people make meaning of the autobiographical events they experience in their lives and relationships. However, people differ in the ways that they react to experiences in their relationships. These differences in how people perceive their relationship highs and lows should similarly influence their narratives about these events.

People hold mental representations—known as working models—of themselves and others which guide their expectations for how others will treat them [9,13,31–33]. The content of these working models is believed to include specific content about events that have transpired in past interactions (e.g., feeling loved, feeling abandoned), as well as influence the information people pay attention to in their social environment [34,35]. Having positive working models of the self and others (e.g., secure attachment) is associated with positive relational outcomes including savoring more of the good in the relationship, as well as more resilience in the face of relationship transgressions [10]. People who are more securely attached are more likely to forgive their partners and prioritize behaviors that enhance rather than undermine relational well-being. People who maintain more positive working models of their partners also tend to see their partner through rose-colored glasses even in the face of interpersonal adversity and conflict. However, those with poor working models of themselves and of others do not see their relationships with the same rosy glow. Insecure attachment styles (e.g., anxious attachment, avoidant attachment) are associated with more negative evaluations of interpersonal transgressions, and less positive evaluations of the partner [10], less trust [11,33,36], difficulty engaging with and providing social support [37], as well as a greater likelihood of relationship dissolution and singlehood (e.g., [38–41]). These divergent patterns of relationship enhancement versus mitigation of the vulnerability inherent in interdependent life are important predictors of relationship satisfaction (positively and negatively respectively). However, most of this work to date has focused on whether people interpret experiences in their relationships as “good” or “bad”. Less is known about how people connect these relationship events with their sense of self, and how people view their own transgressions against a partner (e.g., what does it say about *me* that we had such a bad fight or that my partner would do something so thoughtful for me).

People tend to prioritize the processing of information that confirms—rather than disconfirms or threatens—how they already see themselves [42]. Consequently, the working models of selves and others that people maintain can bias the interpretations people have of interactions so as to maintain their sense of self. Consistent with schematic information processing [43], people who are more securely attached retrieve fewer negative memories about their relationships [44]. Similarly, people who are relatively less avoidantly attached are more likely to use more positive affect when describing their personal love stories, whereas those who are more anxiously attached use less positive affect and more strongly integrate these stories into their sense of self ([27]; see also [45]). On the other hand, being able to recognize one’s own faults is essential for attempting to engage in reparative actions following a transgression. Those who develop “victim” narratives or have more destructive interpretations of relationship conflict are more likely to break up than people who can see the silver lining of these experiences [46,47]. Furthermore, accurately remembering the details of a transgression is important for promoting positive change in the relationship subsequent to the transgression itself [48,49]. Thus, although own transgressions against a partner can threaten positive self-schemas, people who see themselves more positively dispositionally may still be motivated to acknowledge their own role in a transgression for the benefit of the partner and the relationship, while those with more negative self-schemas should be motivated to distance from these experiences or self-denigrate [50].

### Connecting the relationship to the self: Attachment style and narrative construction

Individual differences in attachment insecurity should influence narrative construction following both the high and low moments in a relationship. Those with relatively higher avoidant attachment typically have relatively positive working models of the self, but poor models of others [9]. To preserve their positive self-model, people higher in avoidance may therefore be motivated to avoid constructing narratives with negative self-connections following a transgression in order to prevent the integration of negative information about the self into their identity (e.g., I am someone who upsets others). Those higher in avoidance are also reluctant to build intimacy and closeness in their relationships with others [9]. For example, relatively higher attachment avoidance is associated with a greater likelihood of remembering being *less* supportive to a partner one week after an event, compared to what was reported immediately following the event [10]. Thus, they should make more negative self-connections following relationship high points which are typically characterized by experiences that increase intimacy and dependence (e.g., they’re going to want even more from me in the future).

By contrast, although those relatively higher in attachment anxiety are also insecure in their connections with others, their vulnerability comes from poor working models the self and an expectation that others will abandon them [9]. For those with relatively high anxious attachment, transgressions are often seen as confirmation of their greatest fear that their partner will ultimately abandon them [51]. Thus, they should make more negative, and fewer positive, event connections following transgressions with their partners. However, it is unclear whether they would be motivated to make more, or less, positive and negative event connections for relationship high points. A high point can offer the much-desired confirmatory evidence that they are a valued partner, but this information is also inconsistent with their general self-concept and may actually heighten their vulnerability if such a relationship were to end. For example, those relatively higher in anxious attachment can experience negative emotions following offers of support from their partners, even if they recognize that support is well intended [52].

### Current research

The current research draws from theoretical models of narrative identity development [1], meaning-making [14], and adult attachment theory [9] to investigate individual differences in how people construct narratives of surrounding transgressions and high points in their relationships, and whether these narratives have consequences for relationship satisfaction. People rely on internal narratives to understand themselves and the world around them [1]. Romantic relationships offer a social context through which people find meaning and make sense of themselves and others (e.g., [4,6,7]). However, individual differences influence how people interpret interactions with their social world (e.g., [8]). For example, attachment styles capture differences in people’s working models of themselves, as well as their working models of selves and others (e.g., [9]). The poor working models of the self (anxious attachment) and of others (avoidant attachment) consistently lead to negative relationship outcomes (e.g., [10,53]). Thus, these dispositional biases should affect the way in which people construct narratives and make meaning of events within their relationships. These narratives should in turn inform perceived relationship quality. Insecure attachment styles (i.e., avoidance, anxiety) are robustly associated with lower relationship satisfaction [54]. It is possible that the ways in which insecurely attached individuals develop narratives about the good and bad events they experience in their relationship influence their satisfaction.

In this preregistered study (https://osf.io/4h8ud/?view_only=4570e4c2d44e461f9ba0612052bddc3b) we examine whether the narratives people construct regarding the transgressions and romantic high-points they experience over a one-year period influences their satisfaction with their relationship. The data from this study came from a longitudinal dataset. In wave 2, participants were asked to describe a transgression they had made against their partner since the last survey (i.e., something they had said or done to upset or hurt their partner’s feelings) and a high point in their relationship that had happened in the last 3-months (i.e., something that stands out in their memory as something that was extremely positive). A self-event connection was any point in the transgression and high-point narratives when the participant explicitly linked the event to their understanding of themselves [19], and was classified according to its valence: positive, negative, or neutral/ambiguous, as well as coded for whether it described a change in oneself or revealed a stable and pre-existing aspect of the self.

### Research questions and hypotheses

This paper puts forward the following research questions and hypotheses. Research questions 1 and 2 (RQ1 & RQ1) test cross-sectional hypotheses, and research questions 3 and 4 (RQ3 & RQ4) test longitudinal hypotheses:

***RQ1*.** Do people with relatively greater attachment insecurity construct different narratives when describing transgressions in their romantic relationships than those relatively lower in attachment insecurity? We expected a positive association between anxiety (relative to less anxiety) and use of negative event connections in transgression narratives (**H1a**). We had no directional hypothesis for whether avoidance would be significantly associated with the use of negative event connections in transgression narratives (**H1b**). Furthermore, we expected a negative association between both anxiety, and avoidance, and the use of positive event connections in transgression narratives (**H1c**).

***RQ2*.** Do people with relatively greater attachment insecurity construct different narratives when describing high points in their relationships than those with relatively lower attachment insecurity? We expected a negative association between avoidance (relative to less avoidance) and the use of positive event connections in high point narratives (**H2a**). We had no directional hypothesis for whether anxiety (relative to less anxiety) would be significantly associated with positive event connections in high point narratives (**H2b**). We did not have any a priori expectations that avoidance would be associated with negative event connections in high point narratives (**H2c**), and had no directional hypothesis for whether anxiety would be significantly associated with negative event connections in high point narratives (**H2d**).

***RQ3*.** To what extent does narrative construction of transgressions explain the relationship between anxiety and relationship satisfaction? We expected that the negative association between anxiety and satisfaction would be explained by the tendency to make more negative event connections in their transgression narratives (**H3**).

***RQ4*.** To what extent does narrative construction of high points explain the relationship between avoidance and relationship satisfaction? We expected that the negative association between avoidance and satisfaction would be explained by the tendency to make fewer positive event connections in their high point narratives (**H4**).

## Method

### Design

The data analyzed for this paper was from a prospective longitudinal study called Dating Diaries with individuals in romantic relationships. There were 5 waves of data collection at 3-month intervals across 1-year with rolling participant recruitment from May 2018 to September 2019. In wave 1, participants answered questions about their romantic attachment style, relationship quality, personality traits and character traits. In waves 2–5, participants wrote narratives describing a transgression and a high point that were recently experienced (since the last survey) in their relationship. The rationale for the design and primary research question is reported in [55] and the results are reported in [56]. The current paper involves a secondary analysis regarding the extent to which romantic attachment styles influences the narration of high points and transgressions, and whether such narration explains the relationship between attachment and relationship satisfaction. The analyses involve assessments of attachment in wave 1, the transgression and high point narratives in waves 2–5 and relationship satisfaction at wave 1 and 5. The conceptual questions are different to analyses previously published (blinded for peer review, 2022), and were pre-registered with no analyses for the present study undertaken prior to submission of the pre-registration. Examination of the self-event connection coding was done for [56], but for different research questions; no analyses have been reported on the attachment and relationship satisfaction measures. The pre-registration, data files, and codebook listing all variables at each wave can be found here: https://osf.io/4h8ud. The narrative data cannot be shared publicly due to ethical restrictions as they contain potentially identifiable open-ended responses.

***Addressing Potential Sources of Bias*:** The following steps were taken to address potential sources of bias in this study: 1) Participants were asked to provide narratives of a range of experiences in their romantic relationships, including high points, low points, and transgressions to ensure that they did not make assumptions about the study aims (or to leave them dwelling only on the negative experiences). 2) The coders of the narratives were not involved with the study design or project and underwent training to ensure narrative coding schemes were applied consistently and achieved good inter-rater reliability scores. 4) We pre-registered our design and data analysis plan before analyzing data to ensure it was theory-led. 5) We report all analyses even where these were null findings and point where additional analyses were carried out and were exploratory in nature.

### Participants

In order to be eligible to participate in the study, participants had to be adults aged 18 years or older, who lived in the UK or the USA, and had been in a romantic relationship with their current partner for between 6 months and 2 years at the point of recruitment into the study. As these is a secondary data analysis of an existing dataset, total sample size was determined in line with the original project aims (see [56]), consistent with sample size guidelines for SEM and growth models [57]. Eight hundred and forty-three participants were invited to wave 1, of which 400 participated in wave 1 (47.40% completion rate). Of the 400 who completed wave 1, 264 completed wave 5 (66.00% completion rate, 34.00% attrition rate). Non-completion at each stage was due to participants not responding to the follow-up survey, except for 25 participants who withdrew from the study after wave 1. We excluded participants before conducting any of the models if they did not provide a transgression narrative at wave two. There were 39 participants who reported that they had not committed a transgression against their romantic partner between waves 1 and 2. Listwise deletion were used in SPSS such that participants were excluded if they had missing data on key variables. See Table 1 in [56] for participant response rates and attrition across all five-waves of this study.

These participants were recruited from the UK (*n* = 233) and USA (*n* = 169) via social media, flyers on university campuses and Qualtrics market research panels into an international longitudinal study about personality growth in romantic relationships. Participants reported being female (*n* = 240), male (*n* = 152), transgender (*n* = 3), and non-binary (*n* = 1) with 4 people not providing any information about their gender identity. The mean age (SD) reported at wave 1 was 26.35 (7.54) and ranged from 18 to 75 years. Participants reported being straight or heterosexual (*n* = 315), mostly straight or heterosexual (*n* = 13), gay or lesbian (*n* = 19), bisexual (*n* = 42) and unsure (*n* = 1) with 5 individuals specifying their sexuality in their own terms in an open-text format and 5 individuals not reporting this information. In the UK, most participants reported being White British (*n* = 169), White European (n = 21), Chinese (*n* = 10), Indian (*n* = 6), Black African (*n* = 3), Black Caribbean (*n* = 3), Pakistani (*n* = 3), specified ethnicity using an open text format (*n* = 14) or did not provide this information (*n* = 4). In the USA, most participants reported being “White or Caucasian” (*n* = 119), “Latino or Hispanic” (n = 13), “Black or African American” (*n* = 12), “Asian, Asian American or Pacific Islander” (*n* = 8), “White European” (*n* = 5), “Indian” (*n* = 1), specified ethnicity using an open text format (*n* = 6) or did not provide this information (*n* = 5).

### Procedure

The School of Psychology Ethics Committee at the University of Nottingham (F1030) and Western Washington University (Protocol #18–008) granted approval for the study procedures. In all 5 waves, participants completed an online questionnaire via Qualtrics with self-report questionnaires and written narrative activities about recent experiences in their romantic relationships. The waves were administered at 3-month intervals and the participants were given up to 2-weeks to return the survey. Participants provided informed consent electronically at the start of each survey, and could not continue onto the survey question if they did not provide or declined to consent. Each survey took between 45–60 minutes to complete. At each wave, participants were compensated with £10/$10 Amazon voucher and also entered into a prize draw to win 1 of 4 £10/$10 Amazon vouchers.

### Questionnaires and narrative activities

For brevity, we will only describe the questionnaires and narrative activities that are used in the current analyses, but interested readers can consult the codebook on OSF for the full list of measures.

#### Anxious and avoidant attachment orientations

The 17-item adult attachment questionnaire (AAQ; [53]) in wave 1 assessed individuals’ attachment orientation within their romantic relationships. Participants responded to each item on a ‘1’ (strongly agree) to ‘7’ (strongly disagree) scale. The scale measures two attachment orientations: (1) avoidance (relative to less avoidance)—the extent to which individuals hold negative view of others and avoid intimacy (e.g., *‘I’m nervous when anyone gets too close to me’*) and (2) ambivalence (also known as anxiety; relative to less ambivalence/anxiety)–the extent to which individuals hold a negative self-view and are concerned with abandonment and rejection by their partner (e.g., *‘I often worry my partners don’t really love me’*). After reverse scoring the necessary items, means for each attachment orientation were computed with higher scores indicating heightened avoidant or ambivalent/anxious attachment orientations. The scales had good internal consistency with α = 0.835 for avoidance and α = 0.742 for ambivalence/anxiety.

#### Relationship satisfaction

The 4-item couple satisfaction index (CSI-4; [58]) was administered in waves 1–5. Participants answered questions on a Likert-type responses scales, rating their happiness with their relationship from ‘0’ (extremely unhappy) to ‘6’ (perfect), whether they had a warm and comfortable relationship with their partner from ‘0’ (not at all true) to ‘5’ (completely true), how rewarding and how satisfying their relationship is from ‘0’ (not at all) to ‘5’ (completely). We created a total score for wave 1 and 5 by summating individual items, with higher scores representing higher levels of relationship satisfaction. The measure has excellent internal consistency for wave 1 (α = 0.941) and wave 5 (α = 0.960).

#### Transgression narratives

In wave 2, participants were asked to describe an occasion since the last survey where they had *“said or did something to upset or hurt your [romantic] partner’s feelings”*. They were told that they could report something their partner was unaware of, but they felt *“doesn’t reflect the type of person you want to be in your romantic relationship*.*”* Participants were given a free text box, and asked to describe in full sentences: what happened, when it happened, who was there, what they were thinking and feeling at the time and why this experience was meaningful to them and their relationship. In waves 3–5, we asked participants to write about the transgression discussed in wave 2 while focusing on how they felt about it now. The same narrative instructions from wave 2 were used (i.e., what happened/when/who/thoughts/feelings). Participants were told that if this incident was no longer a meaningful experience to describe, then they could select another incident that had happened since the last survey. We asked participants to indicate if it was the same event as described in wave 2 (yes/no/unsure). As reported in [56] the majority of participants—between 64–70%—reported different and more recent transgressions across waves 3–5, rather than the events reported in wave 2 or the previous wave.

#### High point narratives

In waves 2–5, participants were asked to describe an occasion in their romantic life that had happened in the last 3-months (since the last survey) that *“stands out in your memory as something that was extremely positive”*. Participants were given a free text box, and asked to describe in full sentences: what happened, when it happened, who was there, what they were thinking and feeling at the time, why it was a high point and why this experience was meaningful to them and their relationship.

#### Narrative coding for self-event connections

Transgression and high point narratives for each participant across waves 2–5 were coded for the frequency of self-event connections. A self-event connection is any point in the narrative when a narrator explicitly links the event to their understanding of themselves [19]. Each connection was classified according to the valence: positive, negative, or neutral/ambiguous. Each connection identified was coded for whether it described a change in circumstance or revealed a stable and pre-existing aspect of the self. For example, if a participant wrote: *“my partner abandoned me*, *and it showed me how unlovable I am”* this would be coded as stable negative connection, whereas if another participant wrote: *“I talked to my partner about why she was upset*, *and it helped me understand why she is reactive to this situation”* this would be coded as positive change connection. We also adapted this coding system to capture relationship-event connections where the understanding was based on how the participant behaves in or orients towards relationships (e.g., I have learnt that I need to talk less, and listen more when my partner is upset). The total scores for positive and negative connections across self and relationship were calculated for each wave due to the low frequencies of connections within each subcategory (i.e., stability/change and self/relationship connections). Four undergraduate coders were trained on the coding system, and then completed a reliability phase with 57 narratives, in which they needed to achieve reliability with an expert rater (third author). The reliability was acceptable. The overall kappas across all connections (stable/change, valence, self/relationship) ranged from .74-.78. The kappas for distinguishing self versus relationship connections ranged from .70-.85. The kappas for valence (positive, negative, or neutral) ranged from .76-.81. The kappas for distinguishing change versus stable ranged from .72-.84.

### Data analysis

We used logistic regression on SPSS 28 to test the hypotheses associated with Research Questions 1 and 2. We regressed avoidance and anxiety onto the binary positive and negative event connections variables for the transgression and high points narratives. We used mediation analysis to test the hypotheses associated with Research Questions 3 & 4. We used Mplus8 to regress attachment anxiety/avoidance (X), event connections (M), relationship satisfaction at wave 1 (CV1) and attachment avoidance/anxiety (CV2) onto relationship satisfaction wave 5 (Y) using the bootstrapping procedure.

## Results

### Descriptive statistics

The descriptive statistics for the study variables are presented in Table 1. Table 2 presents the correlations between the study variables. As can be seen from Table 1, our sample self-reported relatively low mean levels of both avoidance and anxiety, and had low frequencies of all event connections in their narratives, regardless of valence. Turning to the correlations (Table 2), as expected, relatively higher avoidance and anxiety were associated with lower relationship satisfaction, but this correlation was observed only in wave 1, not in wave 5. Higher relationship satisfaction in wave 1 was associated with higher satisfaction in wave 5, and avoidance and anxiety were positively associated. Interestingly, avoidance and anxiety were not associated with the use of event connections in the narratives, regardless of valence. However, relationship satisfaction at wave 1 was associated with the use of event connections in narratives. Individuals reporting greater satisfaction made a greater number of positive and negative event connections in their transgression narratives and a greater number of only positive event connections in their high point narratives. Finally, event connections were positively associated, regardless of valence or type of narrative (transgression and high point).

**Table 1:** Descriptive statistics for study variables.

Variable	Mean	Standard Deviation	Sample Size
Anxiety (Wave 1)	3.789	1.075	396
Avoidance (Wave 1)	3.709	1.273	396
Relationship Satisfaction (Wave 1)	15.846	4.091	396
Relationship Satisfaction (Wave 5)	14.699	5.158	262
Negative Connections Transgressions (Waves 2–5)	0.601	1.046	348
Positive Connections Transgressions (Waves 2–5)	0.908	1.202	348
Positive Connections High Points (Waves 2–5)	1.146	1.393	350
Negative Connections High Points (Waves 2–5)	0.120	0.431	350

**Table 2:** Correlations between study variables.

	Anxiety W1	Satisfaction W1	Satisfaction W5	Negative Transgress W2-5	Positive Transgress W2-5	Positive High Points W2-5	Negative High Points W2-5
Avoidance (W1)	.296**	-.148**	-.093	.071	-.042	.066	0.30
Anxiety (W1)	--	-.187**	-.010	.066	-.098	-.012	-.034
Satisfaction (W1)		--	.530**	.118*	.239**	.153**	.067
Satisfaction (W5)			--	-.081	.205**	.105	.046
Negative Transgress (W2-5)				--	.186**	.253**	.380**
Positive Transgress (W2-5)					--	.335**	.125*
Positive High Points (W2-5)						--	.171**
Negative High Points (W2-5)							--

*Variable notes*. Satisfaction w1 = relationship satisfaction in wave 1, Satisfaction w5 = relationship satisfaction in wave 5, negative transgress = negative event connections in transgressions, positive transgress = positive event connections in transgressions, positive high points = positive event connections in high points and negative high points = negative event connections in high points. Connection high points and transgressions were coded across waves 2–5.

** = Significance < .01 (2-tailed) and

* = significance < .05 (2 tailed).

### Examination of pre-reregistered hypotheses

We first checked our data conformed to assumptions before undertaking our pre-registered analyses. However, given the low frequency of event connections, our data seriously violated the assumption of normality. We therefore deviated from our pre-registered data analytic plan to create binary variables for all event connection variables where ‘0’ = no connections made and ‘1’ = one or more event connections made. We analyzed the same research questions, but we used logistic regressions to predict the categorical event connection variables from attachment style.

### Research Question 1

Do those higher in avoidance (relative to lower avoidance) and anxiety (relative to lower anxiety) differ in their construction of narratives about romantic transgressions? We first examined the use of negative event connections. We examined if the data met the assumptions for logistic regression. We found no extreme outliers when examining for cases that exceeded both Cooks and Leverage cut-off scores. There were no issues identified with multicollinearity or the linearity of the logit. We therefore regressed avoidance and anxiety onto the binary negative event connections variable for the transgression narratives. The results are reported in Table 3. There was a positive and significant association between avoidance and negative event connections and the Odds Ratio was greater than 1, indicating that as avoidance increased, relative to low avoidance, the likelihood of the outcome occurring (i.e., using one or more negative event connection) increased by 1.239. The association between anxiety and negative event connections was not significant.

**Table 3:** Logistic regression on negative event connections in transgressions.

			*95% CI for Odds Ratio*		
Included	*b*	*S*.*E*.	Lower	Odds	Upper
Constant	-1.556 [-2.596, -0.648]	.486			
Avoidance	0.214* [.030, 0.412]	.092	1.034	1.239	1.485
Anxiety	0.045 [-.181, .285]	.110	0.844	1.046	1.297

*Note*. R^2^ = .019 (Cox & Snell), .026 (Nagelkerke). Model X^2^ = 6.688, *p<* .05. *< .05. 95% BCa bootstrap confidence intervals based on 1000 samples.

We next examined if those higher in attachment avoidance and anxiety differed in use of positive event connections in transgression narratives. We examined if the data met the assumptions for logistic regression. We found no extreme outliers when examining for cases that exceeded both Cooks and Leverage cut-off scores. There were no issues identified with mutlicollinearity, but linearity of the logit assumption was not met for attachment avoidance. We regressed avoidance and anxiety onto the binary positive event connections variable for transgression narratives. As can be seen from Table 4, there were no significant associations between avoidance or anxiety and the likelihood of use of positive event connections in transgressions.

**Table 4:** Logistic regression on positive event connections in transgressions.

			*95% CI for Odds Ratio*		
Included	*b*	*S*.*E*.	Lower	Odds	Upper
Constant	0.248 [-.647, 1.136]	.448			
Avoidance	.067 [-.094, .255]	.087	0.902	1.070	1.269
Anxiety	-.143 [-.369, .060]	.105	0.867	0.705	1.066

*Note*. R^2^ = .006 (Cox & Snell), .008 (Nagelkerke). Model X^2^ = 2.012, *p* = .366. 95% BCa bootstrap confidence intervals based on 1000 samples.

#### Research Question 2

Do those higher in avoidance (relative to lower avoidance) and anxiety (relative to lower anxiety) differ in their construction of narratives about romantic high points? We first examined the use of positive event connections. We examined if the data met the assumptions for logistic regression. We found no extreme outliers when examining for cases that exceeded both Cooks and Leverage cut-off scores. There were no issues identified with mutlicollinearity, but linearity of the logit assumption was not met for anxiety or avoidance. We regressed avoidance and anxiety onto the binary positive event connections variable for transgression narratives. The results are reported in Table 5. There was a positive and significant association between higher avoidance, relative to low avoidance, and positive event connections and the Odds Ratio was greater than 1, indicating that as avoidance increased, the likelihood of the outcome occurring (i.e., using one or more positive event connection) increased by 1.230. The association between anxiety and positive event connections was not significant.

**Table 5:** Logistic regression on positive event connections in high points.

			*95% CI for Odds Ratio*		
Included	*b*	*S*.*E*.	Lower	Odds	Upper
Constant	-0.261 [-1.191, 0.654]	.451			
Avoidance	0.207* [.033, .381]	.090	1.032	1.230	1.467
Anxiety	-.033 [-.244, .178]	.107	0.785	0.968	1.193

*Note*. R^2^ = .016 (Cox & Snell), .022 (Nagelkerke). Model X^2^ = 5.621, *p =* .060. * < .05. 95% BCa bootstrap confidence intervals based on 1000 samples.

We next examined if those higher in avoidance and anxiety differed in use of negative event connections in high point narratives. We examined if the data met the assumptions for logistic regression. We found no extreme outliers when examining for cases that exceeded both Cooks and Leverage cut-off scores. There were no issues identified with mutlicollinearity or the linearity of the logit. We therefore regressed avoidance and anxiety onto the binary negative event connections variable for the high point narratives. As can be seen from Table 6, there were no significant associations between avoidance or anxiety and the likelihood of use of negative event connections in high point narratives.

**Table 6:** Logistic regression on negative event connections in high points.

			*95% CI for Odds Ratio*		
Included	*b*	*S*.*E*.	Lower	Odds	Upper
Constant	-2.267 [-3.994, -.906]	0.758			
Avoidance	.032 [-.299, .384]	0.154	0.764	1.033	1.396
Anxiety	-.057 [-.471, .336]	.095	0.657	0.945	1.358

*Note*. R^2^ = .000 (Cox & Snell), .001 (Nagelkerke). Model X^2^ = 0.110, *p =* .946. 95% BCa bootstrap confidence intervals based on 1000 samples.

#### Research Question 3

**Fig 1:**
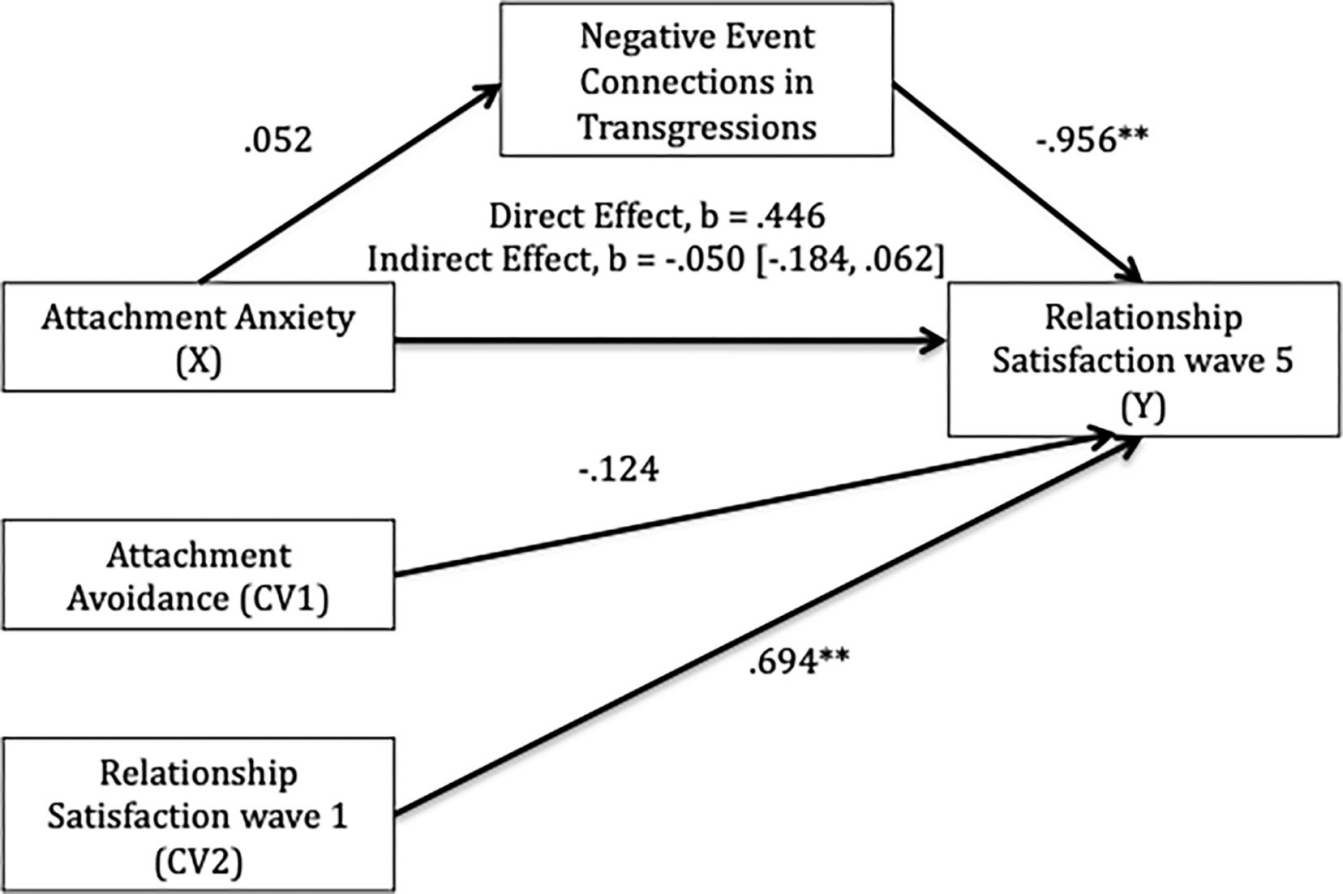
Mediational model predicting relationship satisfaction from attachment style and use of negative event connections in transgression narratives. *Notes*. Values show unstandardized path coefficients. **p < .01.

Does the use of negative event connections in romantic transgression narratives account for the association between anxiety and relationship satisfaction? Specifically, we examined whether the relationship between anxiety and relationship satisfaction at wave 5 was mediated by use of negative event connections in transgression narratives while controlling for relationship satisfaction (at wave 1) and avoidance. Using Mplus8 we regressed anxiety (X), negative event connections (M), relationship satisfaction at wave 1 (CV1) and avoidance (CV2) onto relationship satisfaction wave 5 (Y) with bootstrapping of 5000 samples. We used Mplus 8 to run our specified mediation model given that the negative event connection variable was binary, and PROCESS is unable to handle binary mediators. The indirect pathway between anxiety and satisfaction at wave 1, via negative event connections, was not significant (indirect effect *b* = -.050, *p* = .517, *95% BCa CI* [-.184, .062]; see Fig 1). There was a significant negative association between use of negative event connections and relationship satisfaction at wave 5, such that if individuals used negative event connections they reported lower satisfaction in their relationship at wave 5. Relationship satisfaction reports at wave 1 and wave 5 were positively associated. We had no a priori hypotheses for whether avoidance would be associated with negative event connections in transgression narratives, and therefore did not have mediation analyses planned a priori. We tested these post-hoc given the significant association. However, the indirect effect was not significant for avoidance (*p* = .073).

#### Research Question 4

**Fig 2:**
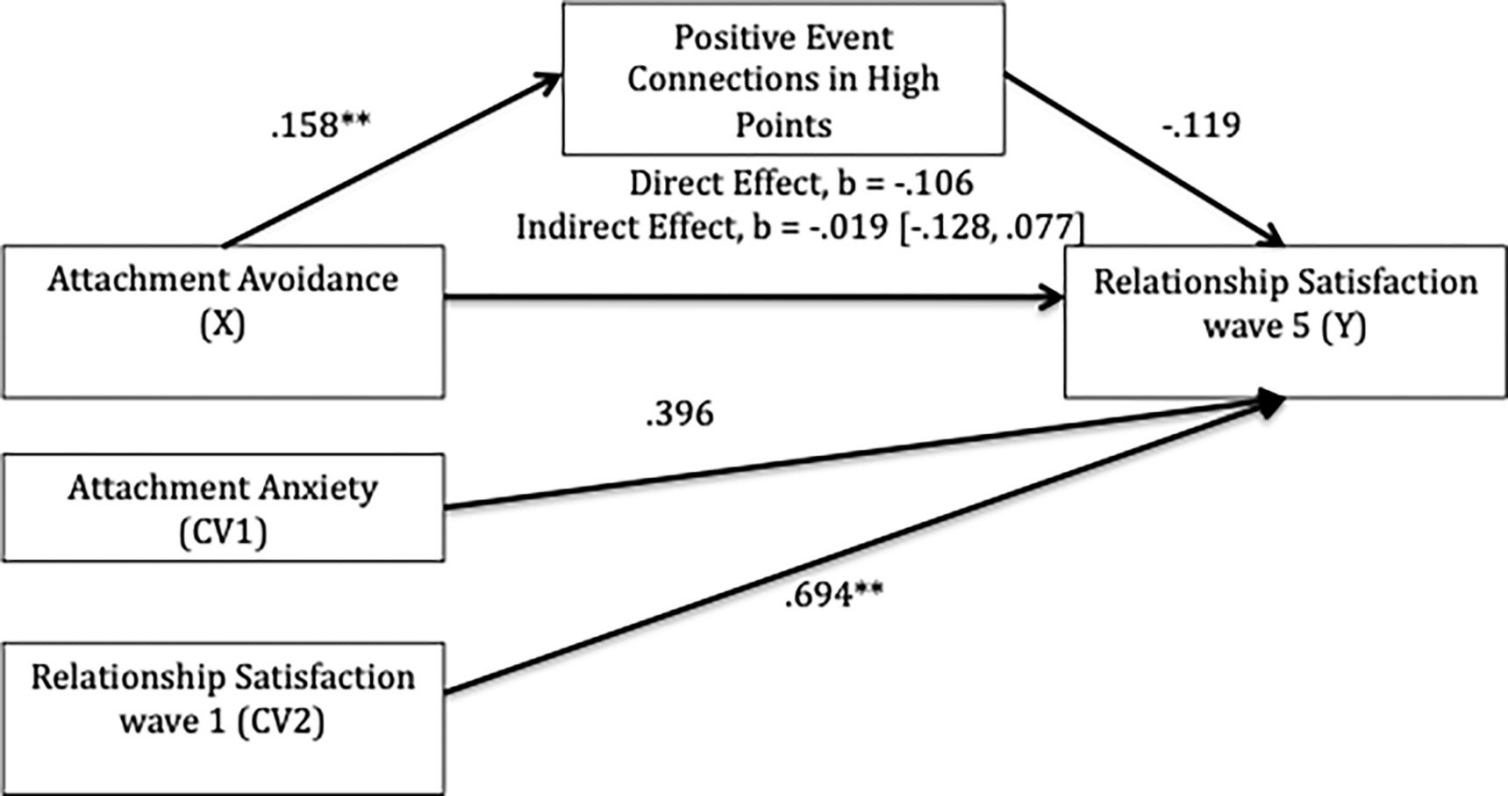
Mediational model predicting relationship satisfaction from attachment style and use of positive event connections in high point narratives. *Notes*. Values show unstandardized path coefficients. **p < .01.

Does the use of positive event connections in romantic high point narratives account for the association between avoidance and relationship satisfaction? Specifically, we examined whether avoidance and relationship satisfaction at wave 5 was mediated by use of positive connections in high point narratives while controlling for relationship satisfaction (at wave 1) and anxiety. Using Mplus8 we regressed attachment avoidance (X), positive connections (M), relationship satisfaction at wave 1 (CV1) and anxiety (CV2) onto relationship satisfaction at wave 5 (Y) with bootstrapping of 5000 samples. The indirect pathway between avoidance and satisfaction at wave 5, via positive connections, was not significant (indirect effect *b* = -.019, *p* = .763, 95% *BCa CI* [-.128, .077]; see Fig 2). There was a significant positive association between avoidance and use of positive event connections, such that those relatively higher in avoidance were more likely to construct high point narratives with positive connections. Relationship satisfaction reports at wave 1 and wave 5 were positively associated.

## General discussion

The aim of this study was to examine whether the narratives people build about themselves based on the good and bad events in their relationships differ as a function of insecure attachment orientations. Additionally, we tested whether differences in the narrative construction of those relatively higher in attachment avoidance and anxiety could account for the negative associations between these insecure attachment and relationship satisfaction. Adult attachment has been reliably linked to relationship outcomes in past research. Insecure attachment shapes interpersonal communication (e.g., reluctance to share emotions for those high in avoidance; focus on own emotions for those high in anxiety; [59]), and undermines relationship satisfaction and trust [11], as well as contributes to psychopathology [60], and makes it difficult for people to recognize their own feelings and emotions [61]. Past work suggests that anxious and avoidance attachment is associated with biased recollections of past events in their relationships (e.g., [10,49]), and respond to both negative and positive experiences with their partners (e.g., [52]). We theorized that these differences would also be evident in the narratives people construct about themselves based on events in their relationships.

When it came to the narratives people constructed about a recent transgression against their partners, greater avoidance was associated with more negative connections in narratives, and not positive connections. By contrast, contrary to our expectations, greater anxiety was neither associated with more negative event connections, nor fewer positive event connections in transgression narratives. For relationship high points, it was again avoidance that was associated with more positive event connections, and not negative connections. By contrast, and contrary to our expectations, anxiety was not associated with either positive or negative connections in narratives about relationship high points.

The associations between attachment anxiety/avoidance and relationship satisfaction were not mediated by the types of event connections people made in relation to relationship transgressions and high points. Unsurprisingly, given the null association between anxiety and negative connections following transgressions, there was no evidence of the negative association between anxiety and relationship satisfaction being mediated by negative connections for transgressions. Likewise, contrary to our hypothesis, despite avoidance being associated with positive connections following high points, there was no evidence of these mediating the association between avoidance and satisfaction.

Although some of these findings are inconsistent with our pre-registered a priori hypotheses, they nonetheless offer interesting insights into how insecurely attached people tell stories about their relationships and link these events back to something meaningful about themselves. Notably, avoidance was more consistently associated with narrative construction following both relationship high points and low points than was anxiety. At first glance, the findings for avoidance may seem inconsistent with the general tendency for avoidantly attached people to disengage from their relationships—and presumably the influence these relationships have on their sense of self. However, these findings may instead help to contextualize other inconsistencies that exist in the broader literature regarding avoidant attachment. For example, avoidance is associated with more positive feelings about the self after being accepted by others, suggesting those who are relatively higher in avoidance still benefit from positive social connection despite their apparent desire to distance from it [62,63]. Although our original hypotheses were centered in a more traditional view of avoidant attachment and their general reticence to engage in social connection (leading us to predict that they would be less inclined to build narratives linking events in their relationships to their self), our findings appear more in keeping with evidence demonstrating that those with more avoidance may claim to not benefit from social connection and yet still derive meaningful information about themselves through these bonds.

Our findings—or lack thereof—for those who are more anxiously attached also raise important questions regarding how they construct narratives about themselves following experiences in their relationships. The current findings suggest that those with more anxiety do not appear to build narratives that connect events in their relationships to themselves. Anxious attachment is often linked with more distorted recollections of past events in the relationship [64], strong ambivalence about their relationships [65], and false memories for past events [49]. People high in anxiety are also more likely to engage in maladaptive metacognitions (e.g., repetitive negative thinking) associated with psychopathology and emotional distress [66]. Psychopathologies have also been linked with disrupted narrative identity development, specifically poorer recall of self-defining events, fewer self-event connections in their narratives, and an inability to extract meaning from these events (e.g., [67]). Thus, although anxious attachment is not a pathological experience, it may share metacognitive processes that interrupt narrative identity development in informative ways. Alternatively, past work suggests that the negative consequences of attachment anxiety are less likely to manifest in benign or neutral contexts, relative to those where their security needs are threatened [68]. The methodology of the current study—asking participants to self-select relevant events from the past few months—may have limited which experiences people selected for. Those more anxiously attached in particular may have selected for more neutral experiences to avoid the threat of more acute experiences that could have elicited the effects of interest for this paper. Finally, attachment anxiety is associated with inconsistent behaviors (e.g., [69]). Thus, their recall and narration of the past may be more dependent on the details of the past than on more characteristic ways of narration (see [70] for a discussion of intra-individual variability in narration).

### Limitations and future directions

Despite this work’s strengths it is not without limitations. First, people were asked to reflect on transgressions and high points that had occurred within the past 2–3 months (i.e., since the last survey). This means that participants had to recall an event that was salient enough for them to remember and stood out among other events. Past research has shown that in addition to having memory biases for events in their relationships, attachment also predicts differences in how these events are remembered over time [10,49]. Whether these distorted recollections are a consequence of shifting perceptions as people tell and retell the stories of these experiences to themselves again and again over time, or whether they are the consequence of different connections made at different time points, they may nonetheless lead to important variance that is not captured through the panel design. Thus, future research might consider using daily diary or experience sampling methods in order to capture more nuanced variability in narrative development, and may therefore be more sensitive to capturing these attachment differences, especially for anxiously attached people who might experience more positive/negative emotion closer to the event [7,48].

The current findings also point to a need for future research to explore how narrative identity development through relationship experiences contributes to relationship satisfaction. Relationship satisfaction represents our global assessment of how well our relationship is meeting our needs and expectations, and whether the positive experiences in our relationships outweigh the negatives [12]. Despite no evidence of mediation between insecure attachment and relationship satisfaction, negative event connections in transgression narratives *were* significantly and negatively associated with relationship satisfaction in the current study. Furthermore, past work has found that daily variability in satisfaction is associated with future relationship dissolution [71]. People who are more inclined to make negative event connections in their transgression narratives may experience dips in relationship satisfaction and this variability may contribute to poorer relationship outcomes in the future. Thus, future research should investigate the links between narrative identity development following relationship experiences and trajectories of relationship satisfaction over time.

Another limitation of the current research was that it was restricted to examining these processes in established, committed relationships. Insecure attachment is associated with a higher propensity for singlehood [40,41,72]. Thus, an insecurely attached person who is single may be qualitatively different than one who is willing or able to maintain a long-term relationship, and may therefore differ in the types of narratives they tell about themselves and their relationships. Relatedly, the findings also suggest that relationship dissolution—often the culmination of relationship disputes and hurt feelings [73]—may be particularly impactful on the narrative identity development for avoidantly attached people. Our findings suggest that avoidance is associated with more negative narratives about the self after a transgression. Avoidantly attached people may therefore have particularly negative narratives about themselves after a breakup. These narratives could contribute to the belief that connection and intimacy are not possible, which lead to attachment system deactivation, and missed opportunities to start new relationships [41]. Thus, future research examining narrative identity development across relationship status, both cross-sectionally and over time, and the moderating role of attachment could help further bridge the literature on the shared and unique experiences of single versus partnered people.

## Conclusion

The stories we tell about ourselves are an important tool for building a coherent identity. Romantic relationships play an important role in narrative identity development by providing a context in which meaningful good and bad experiences occur that we can link to ourselves. Attachment avoidance constrains narrative development, with greater avoidance leading to more negative stories about the self following a transgression against a partner, and more positive stories about the self when the relationship is going well.
